# Effects of elevated temperature and CO_2_ on intertidal microphytobenthos

**DOI:** 10.1186/s12898-015-0043-y

**Published:** 2015-04-01

**Authors:** Paulo Cartaxana, Sónia Vieira, Lourenço Ribeiro, Rui JM Rocha, Sónia Cruz, Ricardo Calado, Jorge Marques da Silva

**Affiliations:** Department of Biology, Marine Biological Section, University of Copenhagen, Strandpromenaden 5, DK-3000 Helsingør, Denmark; MARE – Marine and Environmental Sciences Centre, Faculdade de Ciências da Universidade de Lisboa, Campo Grande, 1749-016 Lisboa, Portugal; Centro de Biodiversidade, Genómica Integrativa e Funcional (BioFIG), Faculdade de Ciências da Universidade de Lisboa, Campo Grande, 1749-016 Lisboa Portugal; Departamento de Biologia & CESAM – Centro de Estudos do Ambiente e do Mar, Universidade de Aveiro, Campus de Santiago, 3810-193 Aveiro, Portugal

**Keywords:** Microphytobenthos, Diatoms, Temperature, Carbon dioxide, Photosynthesis, Climate change

## Abstract

**Background:**

Microphytobenthos (MPB) are the main primary producers of many intertidal and shallow subtidal environments. Although these coastal ecosystems are particularly vulnerable to anthropogenic activities, little is known on the effects of climate change variables on the structure and productivity of MPB communities. In this study, the effects of elevated temperature and CO_2_ on intertidal MPB biomass, species composition and photosynthetic performance were studied using a flow-through experimental life support system.

**Results:**

Elevated temperature had a detrimental effect on MPB biomass and photosynthetic performance under both control and elevated CO_2_. Furthermore, elevated temperature led to an increase of cyanobacteria and a change in the relative abundance of major benthic diatom species present in the MPB community. The most abundant motile epipelic species *Navicula spartinetensis* and *Gyrosigma acuminatum* were in part replaced by tychoplanktonic species (*Minidiscus chilensis* and *Thalassiosira* cf. *pseudonana*) and the motile epipelic *Nitzschia* cf. *aequorea* and *N.* cf. *aurariae*. Elevated CO_2_ had a beneficial effect on MPB biomass, but only at the lower temperature. It is possible that elevated CO_2_ alleviated local depletion of dissolved inorganic carbon resulting from high cell abundance at the sediment photic layer. No significant effect of elevated CO_2_ was detected on the relative abundance of major groups of microalgae and benthic diatom species.

**Conclusions:**

The interactive effects of elevated temperature and CO_2_ may have an overall detrimental impact on the structure and productivity of intertidal MPB, and eventually in related ecosystem services.

**Electronic supplementary material:**

The online version of this article (doi:10.1186/s12898-015-0043-y) contains supplementary material, which is available to authorized users.

## Background

Microphytobenthos (MPB) are phototrophic communities that constitute the main primary producers of intertidal and shallow subtidal ecosystems [1,2]. Usually diatom-dominated, MPB mediate nutrient cycling, enhance benthic-pelagic coupling and act as efficient sediment stabilizers [3,4]. Although coastal ecosystems are particularly vulnerable to climate change, little is known on the effects of variables such as elevated temperature or CO_2_ availability on MPB productivity and related ecosystem services.

Within specific ranges, increased temperature generally results in higher metabolic activity and thus increased growth rates. Accordingly, photosynthesis and productivity of cultured benthic diatoms [5,6] and natural MPB communities [7,8] have been shown to increase with transient high temperature. However, much less is known on the effect of temperature changes on MPB at longer time-scales. A gradual transition from a phototrophic to a heterotrophic-dominated benthic community with increasing temperature has been previously reported for intertidal and subtidal systems [9,10]. Hicks et al. [11] found a detrimental effect of higher temperatures on MPB biomass in a 7-day experiment.

Photosynthesis in marine diatoms is generally not limited by inorganic carbon availability due to the operation of carbon concentrating mechanisms (CCMs) e.g. [12,13]. However, a few studies on diatoms as part of highly productive MPB biofilms suggest limitation of photosynthesis by inorganic carbon availability. Admiraal et al. [14] found that the diffusion of inorganic carbon limited the productivity of dense unialgal mats of the diatom *Navicula salinarum*. Addition of HCO_3_^−^ was also found to increase photosynthetic rates of MPB natural communities in subtidal sand [15] and in intertidal muddy sediments (Vieira S., unpublished data). On the other hand, at a longer time-scale, Hicks et al. [11] found no significant increase on MPB biomass in subtidal mesocosms under increased atmospheric CO_2_ levels.

Several authors have stressed the importance of studying the combined effects of different environmental drivers on ecosystem functioning e.g. [16]. Recent studies have shown interactive negative effects of increased temperature and CO_2_ in marine phytoplankton e.g. [17,18]. Elevated temperatures resulting from global climate change as low as 2–3°C can be expected to affect microalgal species differently, causing increased metabolic activity and growth of some species while pushing others beyond their temperature optima, thus changing species composition [19]. To our knowledge, only Hicks et al. [11] addressed the interactive effects of elevated atmospheric CO_2_ concentrations and temperature on MPB biomass, using a nontidal experimental mesocosms. In this work, the combined effects of elevated temperature and CO_2_ on MPB biomass, photosynthetic performance and species composition were studied on an intertidal system using a flow-through experimental life support system with a simulated tidal regime.

## Methods

### Sediment sampling and set-up

The sediment surface layer (approximately the first 2 cm) was collected during a summer low tide at Alcochete intertidal flats, Tagus estuary, Portugal (38°44'45''N, 08°59'04''W). Sediment was transported in refrigerated containers to the laboratory, homegeneized and placed inside microcosms in a flow-through experimental life support system (ELSS), forming a layer of 6 cm.

Induction of MPB cell distribution within the sediment profile was achieved by exposing the sediment to an irradiance of 70 μmol photons m^−2^ s^−1^ for *ca.* 24 h. Establishment of the MPB surface biofilm was assessed by measuring the normalized difference vegetation index (NDVI, see below). Once the MPB surface biofilm was established, all microcosms were subjected to the initial conditions of temperature and pH (18°C, pH 8.0). After 24 h at these conditions, four different treatments were started and the experiment run for 11 days: 1) 18°C and pH 8.0; 2) 24°C and pH 8.0; 3) 18°C and pH 7.4; and 4) 24°C and pH 7.4. Four microcosms were used for each treatment (with a total of 16 microcosms being used in the whole experiment).

The temperatures were chosen within the summer variation range of the study site and corresponded to mean high tide (18°C) and mean diurnal low tide (24°C) sediment temperatures [20]. The pH of the sediment interstitial water was 8.0, while a pH drop of 0.6 units (pH 7.4) was chosen on the basis of the Intergovernmental Panel on Climate Changes [21] maximum projections for the change in global ocean surface pH (~0.4 units) in 2100, together with possible increased acidification caused by upwelling of anthropogenic CO_2_-enriched water in coastal systems [22].

### Experimental life support system (ELSS)

A flow-through ELSS was used, as described in detail by Coelho et al. [23]. The ELSS consisted of 16 independent microcosms (glass tanks - 28 cm length x 25 cm height x 12.4 cm width) with a maximum functional water volume of approximately 7 L (see Additional file 1: Figure S1). The ELSS was equipped with 4 full spectrum fluorescent tubes (AquaLight, T5/54 W/10000K, Bramsche, Germany) and set to 6 h light–18 h dark cycle with an irradiance at sediment surface of 70 μmol photons m^−2^ s^−1^.

The ELSS was operated with one daily tide. Saltwater was prepared in two reservoirs (230 L each) by mixing freshwater purified by a reverse osmosis unit (Aqua-win RO-6080) with a commercially available marine salt mixture (Tropic Marin Pro Reef salt – Tropic Marine, Germany) to a final salinity of 30. The water for tidal cycles was prepared 24 h before use. To simulate high tide, saltwater was pumped from the respective reservoir using a submersible pump (Aquabee UP 3000) through an independent pipeline system of polyvinyl chloride (PVC) tubes into each microcosm. The saltwater flow rate was manually controlled by a PVC valve located above each microcosm. The saltwater input was stopped when the water layer reached *ca.* 15 cm. High tide started after 15 min of the onset of the dark period. To simulate low tide, outflow submersible pumps (Rena flow 400 C) were used in each microcosm, operated using digital timers. These pumps were positioned inside a PVC cylinder and protected with a mesh screen to avoid clogging. The water was discharged using a common outflow pipe. Low tide started 15 min before the period of light exposure.

The microcosms in the ELSS were partially submerged into two main water-bath tanks. One tank was set to 18°C, the water was continuously pumped by a canister filter pump (SunSun HW-302) through a cooler equipped with a thermostat (Teco TR10) with a flow rate of 1000 L h^−1^. The other tank was equipped with two submersible 200 W heaters with thermostats (Rena Cal 200) set to increase water temperature to 24°C.

Water pH was manipulated by acidifying the water stocked in the saltwater reservoirs by bubbling CO_2_ through a diffuser. The diffuser operated with a water pump (Aquabee UP 3000) to maximize CO_2_ gas mixing in saltwater. CO_2_ addition was controlled with a feedback system that included a combination of a pH electrode connected to a controller (V^2^ control pH controller, Tropical Marine Centre, Bristol, UK) and a pressure regulator with an integrated solenoid valve (V^2^ pressure regulator pro, Tropical Marine Centre, UK). The digital display of the controller allowed visualization of actual pH in the saltwater reservoir and pH monitoring with the pH electrode. The controller opened the solenoid valve whenever pH rose above the set value; CO_2_ was then injected until water pH returned to the pre-set value.

### MBP biomass

MPB biomass was estimated daily and non-intrusively in each microcosm by calculating NDVI. Daily measurements of spectral reflectance as well as Pulse Amplitude Modulated (PAM) fluorescence (see below) were done in all microscosms during low tide, starting after 90 min of light exposure to ensure that the sediment was in the same conditions regarding diatom migration and biofilm establishment. Reflectance spectra were measured over a 350–1000 nm bandwidth with a USB4000 (Ocean Optics, USA) with a VIS-NIR optical configuration connected to a 400 μm diameter fiber optic (QP400-2-VIS/NIR, Ocean Optics, USA). The light spectrum reflected from the sample was normalized to the spectrum reflected from a clean polystyrene plate. A reflectance spectrum measured in the dark was subtracted to both spectra to account for the dark current noise of the spectrometer. The fiber optic was positioned perpendicularly to the sediment surface and both sample and reference spectra were measured under a constant irradiance of 70 μmol photons m^−2^ s^−1^. NDVI was calculated as (R750 − R675) / (R750 + R675), where R750, R675 and R636 represented the average diffusive reflectance in the intervals of 749.73–750.39 nm, 674.87–675.55 nm and 635.71–636.40 nm, respectively [24].

Additionally, MPB biomass was calculated using HPLC chlorophyll *a* (Chl *a*) analysis at the beginning (T0) and at the end of the experimental period (T11). Invasive sampling for Chl *a* determination was done because previous studies have indicated NDVI saturation for high MPB biomass [24,25]. Sampling for Chl *a* was performed after spectral reflectance and PAM fluorescence measurements. For Chl *a* analysis, one sediment minicore (diameter 1.1 cm) was collected per microcosm at the beginning of the experiment (T0) using a plastic corer. The sediment surface (0 – 2 mm) was pooled in groups of 4 to obtain 4 mixed sediment samples. At the end of the experiment (T11), three minicores were collected per microcosm and the sediment pooled to obtain a total of 16 samples, one per microcosm. Sediment samples were immediately frozen in liquid nitrogen and stored at −80°C. Before analysis, samples were freeze-dried and extracted with 95% cold buffered methanol (2% ammonium acetate) for 15 min at −20°C, in the dark. Samples were sonicated (1210, Bransonic, USA) for 30 s at the beginning of the extraction period. Extracts were filtered (Fluoropore PTFE filter membranes, 0.2 μm pore size) and immediately injected in a high performance liquid chromatographer (HPLC; LC10AVP, Shimadzu, Japan) equipped with a photodiode array (SPD-M10AVP) detector [26]. Chromatographic separation was carried out using a C18 column for reverse phase chromatography (Supelcosil; 25 cm long; 4.6 mm in diameter; 5 mm particles) and a 35 min elution programme. The solvent gradient followed Kraay et al. [27] with a flow rate of 0.6 mL min^−1^ and an injection volume of 100 μL. Chl *a* was identified from absorbance spectrum and retention time and concentrations calculated from the signals in the photodiode array detector. Calibration of the Chl *a* peak was performed using a commercial pigment standard from DHI (Institute for Water and Environment, Denmark).

### MPB photosynthetic parameters

Measurement of MPB photosynthetic parameters were carried out in each microcosm using a Diving-PAM Fluorometer (Walz, Effeltrich, Germany). The distance between the fluorometer fiber optic and the surface of sample was kept constant at 2 mm during all measurements. Maximum quantum yield of photosystem (PS) II (*F*_v_/*F*_m_) was determined daily in each microcosm by calculating (*F*_m_ – *F*_o_)/*F*_m_, where *F*_m_ and *F*_o_ are, respectively, the maximum and the minimum fluorescence of dark-adapted samples [28]. *F*_v_/*F*_m_ gives a robust indication of the maximum efficiency of photosynthesis. Dark adaptation period was restricted to 2 min to reduce the possibility of inducing downward vertical migration of the epipelic MPB [29].

On specific days (T0, T6 and T11), rapid light-response curves (RLC) were carried out in all microcosms to assess MPB photosynthetic activity over a wide range of ambient light intensities [30]. For the construction of RLC, the samples were exposed to 8 intensities of actinic light increasing from 38 to 616 μmol photons m^−2^ s^−1^ (38, 55, 81, 122, 183, 262, 367 and 616 μmol photons m^−2^ s^−1^). Each irradiance step was 10 s; the saturation pulse intensity had duration of 0.6 s and an intensity of 8,000 μmol photons m^−2^ s^−1^. RLC were constructed by calculating, for each level of actinic light, the effective quantum yield of PSII (Δ*F*/*F*_m_′) and the relative electron transport rate (r*ETR*) from the delivered actinic irradiance (*E*) by r*ETR* = *E* x Δ*F*/*F*_m_′ [30]. The light response was characterized by fitting the model of Platt et al. [31] to r*ETR* vs *E* curves and by estimating the initial slope of the light curve *α* (light utilization coefficient), *ETR*_max_ (maximum r*ETR*) and *E*_k_ (light saturation parameter), where *E*_k_ = *ETR*_max_ /*α*. The model was fitted iteratively using MS Excel Solver.

### MPB community analysis

Surface sediment samples to determine the composition of the MPB community were collected as described for Chl *a* analysis and stored in a 2.5% glutaraldehyde solution at 4°C. Cells were extracted from the sediment following an isopycnic separation technique using silica sol Ludox HS-40 that separates the organic material from mineral particles and is, thus, able to remove microorganisms (e.g. MPB) from the sediment [32]. Cell counts of MPB were made in a Sedgwick-Rafter cell counting chamber (50 μL of each extract) on an Olympus BX50 optical microscope (Olympus Corporation, Tokyo, Japan) at a 400x magnification. Between 3 and 9 horizontal transects (1300 – 8500 individual cells) were made, the cells counted separated into major MPB taxonomical groups (i.e. diatoms, euglenids, dinoflagellates and cyanobacteria) and the relative percentage determined.

Diatom analysis was conducted after cleaning the diatom valves of organic material. A subsample of 750 μL of extract was oxidized with 5–7 mL of hydrogen peroxide (30%) at 90°C for at least 4 h. Permanent slides, mounted in Naphrax^TM^ (Northern Biological Supplies Ltd., Ipswich, UK), were made for each sample. Phase and differential interference contrast optical microscopy were used to identify and count diatoms at a magnification of 1,000x. For each slide, a minimum of 400 valves were counted and identified to the species level, following Ribeiro [32] and references therein.

### Statistical analysis

The existence of significant differences was tested using two-way repeated measurements ANOVA (NDVI, *F*_v_/*F*_m_, and RLC parameters) or two-way ANOVA (Chl *a* and MPB major group relative abundance) for the effects of temperature (18 and 24°C) and pH (7.4 and 8.0). Multiple comparisons were performed using Tukey HSD. Bonferroni correction was applied to p values of multiple tests on correlated variables (NDVI and Chl *a*; *ETR*_max_, *α* and *E*_k_; relative abundance of diatoms and cyanophytes). Statistical analyses were carried out using Statistica 10 (StatSoft Inc., USA).

Diatom community structure was analysed with non-parametric multivariate tools using PRIMER® 6 software package (PRIMER-E, Plymouth, UK). The species abundance matrix was previously standardized and root-transformed and used in all multivariate routines. Bray-Curtis coefficients [33] were used to compute the similarity or dissimilarity distances between samples. A similarity-based ANOSIM permutation test, with a 2-way crossed layout [34], was performed to test if there were any statistically differences between groups of samples, namely, between temperature or pH treatments. A classification analysis (CLUSTER), which uses hierarchical agglomerative clustering of the samples and group-average linking [35], was also performed. During the dendrogram construction statistical significance of every cluster node was tested by the SIMPROF routine [36]. The SIMPROF is an *a posteriori* permutation test of the null hypothesis that the set of samples below a given node does not show any multivariate structure, which are then represented by dashed lines. Species mainly responsible for possible differences between treatments were determined using SIMPER analysis [34].

## Results

### MPB biomass

**Figure 1:**
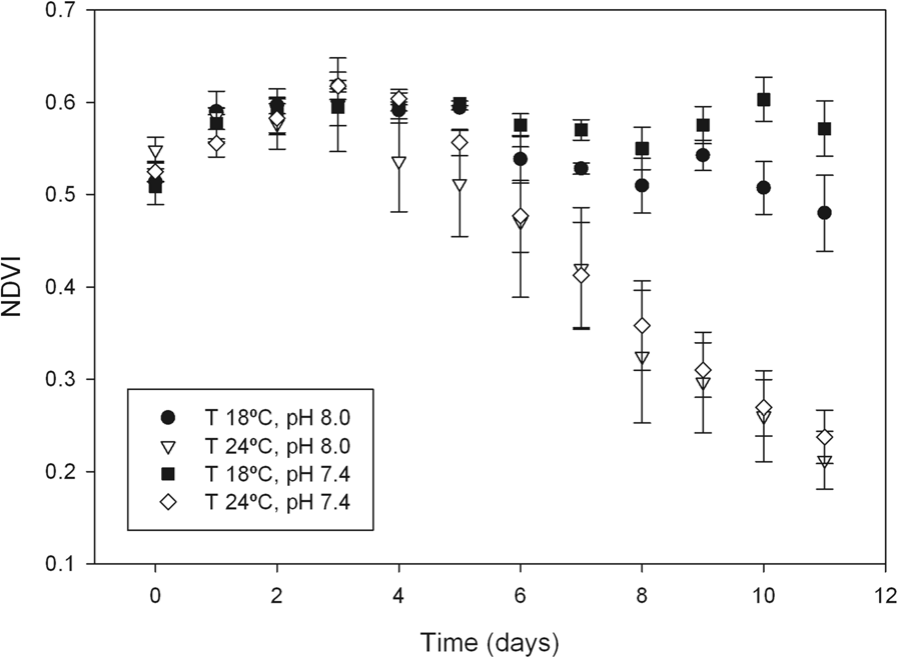
**Microphytobenthos NDVI under control and elevated CO**_**2**_ **and temperature.** Changes in normalized difference vegetation index (NDVI, mean ± standard error, n = 4) of an intertidal sediment during an 11-day period under different temperatures and pH. T 18°C, pH 8.0: Temperature = 18°C, pH = 8.0; T 24°C, pH 8.0.Temperature = 24°C, pH = 8.0; T 18°C, pH 7.4.Temperature = 18°C, pH = 7.4; T 24°C, pH 7.4: Temperature = 24°C, pH = 7.4

There was a significant effect of temperature on NDVI measured along the experimental time period (F_11,132_ = 28.172, p < 0.001), but no significant effect of pH (F_11,132_ = 1.131, p = 0.686; Figure 1). There was no significant interaction between the categorical factors (temperature and pH; F_11,132_ = 0.937, p = 1.000; Figure 1). Between day 0 and 3, NDVI increased slightly in all treatments, followed by a decrease in the microcosms at 24°C, reaching values of *ca.* 0.2 after 11 days (Figure 1). At 18°C, NDVI was relatively constant throughout the experiment (ranging between 0.5 and 0.6).

**Figure 2:**
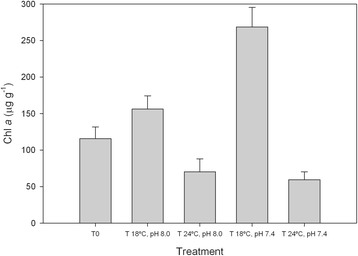
**Microphytobenthos Chl** ***a*** **under control and elevated CO**_**2**_ **and temperature.** Chlorophyll *a* concentration (Chl *a*, mean ± standard error, n = 4) of an intertidal sediment (0–2 mm) at the beginning of the experiment (T0) and after 11 days under different temperatures and pH. T 18°C, pH 8.0: Temperature = 18°C, pH = 8.0; T 24°C, pH 8.0.Temperature = 24°C, pH = 8.0; T 18°C, pH 7.4.Temperature = 18°C, pH = 7.4; T 24°C, pH 7.4: Temperature = 24°C, pH = 7.4

There was a significant interaction between temperature and pH on Chl *a* concentrations (F_1,12_ = 10.329, p = 0.015; Figure 2). Chl *a* concentrations were higher at 18°C than at 24°C, similar to what was observed with NDVI. On the other hand, at 18°C, Chl *a* concentrations were significantly higher at pH 7.4 than pH 8.0, reaching concentrations of 268 ± 53 μg g^−1^ (p = 0.014; Figure 2). No significant differences were observed between pH 7.4 and 8.0 at 24°C (p = 1.000; Figure 2).

### MPB photosynthetic parameters

**Figure 3:**
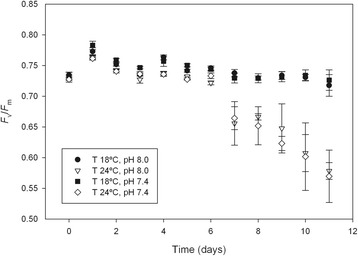
**Microphytobenthos** ***F***_**v**_ **/** ***F***_**m**_ **under control and elevated CO**_**2**_ **and temperature.** Changes in maximum efficiency of photosystem (PS) II (*F*_v_/*F*_m_, mean ± standard error, n = 4) of an intertidal sediment during an 11-day period under different temperatures and pH. T 18°C, pH 8.0: Temperature = 18°C, pH = 8.0; T 24°C, pH 8.0.Temperature = 24°C, pH = 8.0; T 18°C, pH 7.4.Temperature = 18°C, pH = 7.4; T 24°C, pH 7.4: Temperature = 24°C, pH = 7.4.

There was a significant effect of temperature on maximum efficiency of PSII (*F*_v_/*F*_m_) measured along the experimental time period (F_11,132_ = 11.560, p < 0.001), but no significant effect of pH (F_11,132_ = 0.170, p = 0.998; Figure 3). At 18°C, *F*_v_/*F*_m_ was relatively constant throughout the experiment (*ca.* 0.73), although a slight increase was observed between day 0 and 1 for all treatments. At 24°C, *F*_v_/*F*_m_ decreased from day 7, reaching significantly lower values (<0.58) at the end of the experiment.

**Figure 4:**
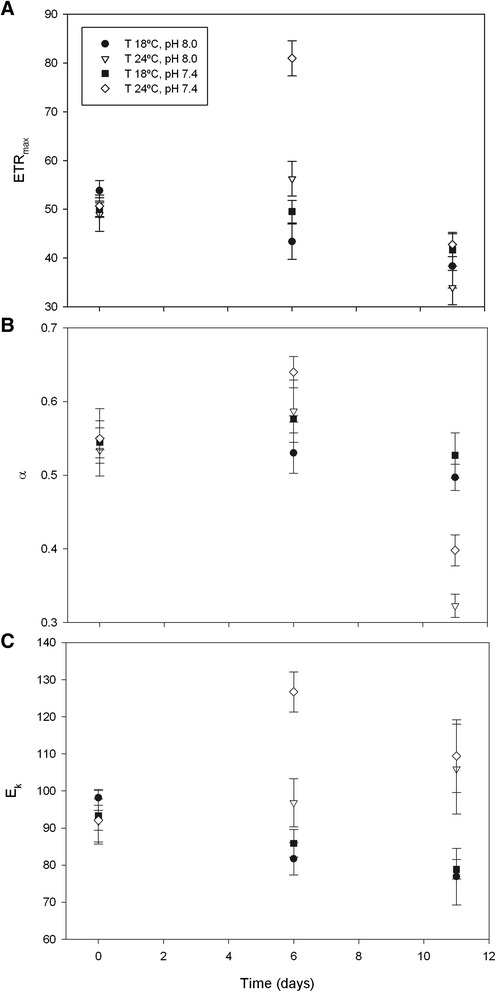
**Microphytobenthos RLC parameters under control and elevated CO**_**2**_ **and temperature.** Changes in relative maximum electron transport rate (r*ETR*_max_, **A**), light utilization coefficient (*α*, **B**) and light saturation parameter (*E*_k_, **C**) (mean ± standard error, n = 4) of an intertidal sediment after 0, 6 and 11 days under different temperatures and pH. T 18°C, pH 8.0: Temperature = 18°C, pH = 8.0; T 24°C, pH 8.0.Temperature = 24°C, pH = 8.0; T 18°C, pH 7.4.Temperature = 18°C, pH = 7.4; T 24°C, pH 7.4: Temperature = 24°C, pH = 7.4.

There was a significant effect of both temperature (F_2,24_ = 21.824, p < 0.001) and pH (F_2,24_ = 7.763, p = 0.008) on *ETR*_max_ measured along the experimental time period (Figure 4A). After 6 days, photosynthetic electron transport capacity was significantly higher at 24°C and pH 7.4, when compared to other treatments (in all cases p < 0.001). At beginning (T0) or at the end of the experimental period (T11), differences in *ETR*_max_ were not significant. For *α*, there was a significant effect of temperature (F_2,24_ = 19.461, p < 0.001), but no significant effect of pH (F_2,24_ = 1.136, p = 1.000; Figure 4B). After 11 days, light utilization coefficient was significantly lower at 24°C than at 18°C (p < 0.001). Regarding *E*_k_, there was a significant effect of temperature (F_2,24_ = 11.827, p < 0.001), but no significant effect of pH (F_2,24_ = 3.339, p = 0.158; Figure 4C), reflecting the trends observed for *ETR*_max_ and *α*. No significant interactions between the categorical factors (temperature and pH) were observed for any of the photosynthetic parameters analysed (lowest p = 0.669).

### MPB taxonomic composition

**Figure 5:**
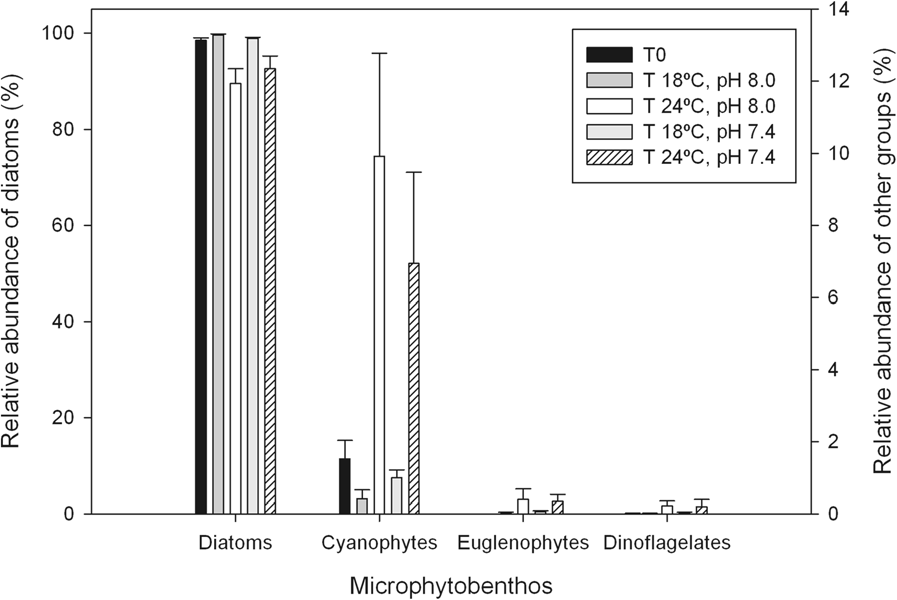
**Relative abundance of major groups of microphytobenthos under control and elevated CO**_**2**_ **and temperature.** Relative abundance (%, mean ± standard error, n = 4) of major groups of microphytobenthos (diatoms, cyanobacteria, euglenophytes and dinoflagelates) of an intertidal sediment (0–2 mm) at the beginning of the experiment (T0) and after 11 days under different temperatures and pH. T 18°C, pH 8.0: Temperature = 18°C, pH = 8.0; T 24°C, pH 8.0.Temperature = 24°C, pH = 8.0; T 18°C, pH 7.4.Temperature = 18°C, pH = 7.4; T 24°C, pH 7.4: Temperature = 24°C, pH = 7.4.

There was a significant effect of temperature on the relative abundance of MPB major groups (F_1,12_ = 16.035, p = 0.003 for diatoms and F_1,12_ = 16.296, p = 0.003 for cyanophytes; Figure 5), while pH had no significant effect (F_1,12_ = 0.348, p = 1.000 and F_1,12_ = 0.392, p = 1.000, respectively; Figure 5). No significant interactions between the categorical factors (temperature and pH) were observed (lower p = 0.739). Although diatoms were the dominant group of the MPB community at the beginning and at end of all experimental treatments, diatom relative abundance was lower after 11 days under the higher temperature (99.2 ± 0.2% at 18°C compared to 91.0 ± 2.0% at 24°C). The decrease in diatoms at 24°C was associated with an increase in the relative abundance of cyanobacteria (0.70 ± 0.2% at 18°C and 8.40 ± 1.9% at 24°C). The contribution of euglenophytes and dinoflagelates to the MPB community was minor, representing in all cases less than 1% of relative abundance.

**Figure 6:**
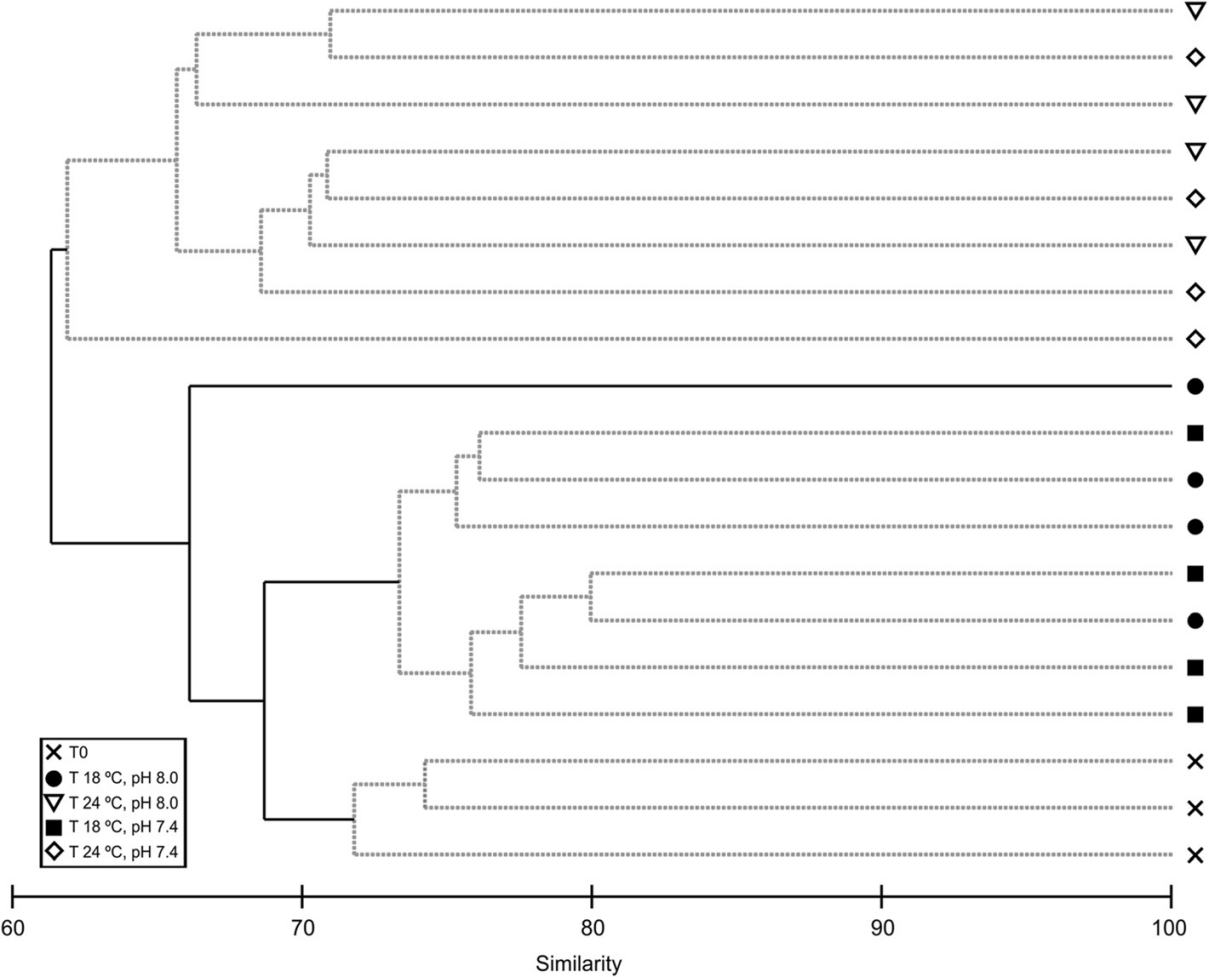
**CLUSTER analysis of diatom assemblage structure under control and elevated CO**_**2**_ **and temperature.** Dendrogram for hierarchical clustering using group-average linking of Bray–Curtis similarities of diatom abundance of an intertidal sediment (0–2 mm) at the beginning of the experiment (T0) and after 11 days under different temperatures and pH. T 18°C, pH 8.0: Temperature = 18°C, pH = 8.0; T 24°C, pH 8.0.Temperature = 24°C, pH = 8.0; T 18°C, pH 7.4.Temperature = 18°C, pH = 7.4; T 24°C, pH 7.4: Temperature = 24°C, pH = 7.4. Dashed lines indicate groups of samples not separated (at p < 0.05) by SIMPROF.

Concerning diatom assemblages, a total of 120 diatom taxa were identified (97 to the species level, see Additional file 2: Table S1), varying between 24 and 57 per sample. Significant differences in diatom assemblage structure were found between 24°C and 18°C (two-way crossed ANOSIM test: R = 0.667, p < 0.001), whereas differences between pH treatments were not significant (R = −0.063, p = 0.713). The CLUSTER analysis of the assemblage structure also showed that there were significant differences between the two incubation temperatures but not between pH (Figure 6), in spite of relatively high levels of similarity (i.e. between 60 and 80%). Samples from the microcosms at 24°C separated significantly from the microcosms at 18°C at 61.3% level of similarity (SIMPROF test: π = 2.24, p < 0.001). Samples collected at the beginning of the experiment (T0) separated significantly at 68.7% of level of similarity (SIMPROF test: π = 0.84, p = 0.019) from the samples collected at the end of the experimental period in the microcosms at 18°C. One of the samples of the 18°C group also separated significantly (SIMPROF test: π = 1.15, p = 0.002) from the rest early in the dendrogram, possible because it registered lower diversity and the highest relative abundance (68%) of *Navicula spartinetensis*. There was no subsequent significant multivariate pattern in the CLUSTER analysis (noted by the grey dotted lines in Figure 6).

Diatom assemblages were taxonomically similar (Additional file 2: Table S1), with an average of 98% of cumulative relative abundance of shared species. Nevertheless, SIMPER analysis was able to detect slight differences in species relative abundance, responsible for the significant dissimilarities in assemblage structure between microcosm temperatures, as shown by ANOSIM and CLUSTER analysis. In this way, assemblages incubated at 18°C had higher abundances of *N. spartinetensis* and *Gyrosigma acuminatum*, whilst in assemblages at 24°C these two motile epipelic species were in part replaced by tychoplanktonic species (i.e. *Minidiscus chilensis*, *Thalassiosira* cf. *pseudonana*) and the motile epipelic *Nitzschia* cf. *aequorea* and *N.* cf. *aurariae* (Additional file 2: Table S1).

## Discussion

In the present 11-day study, elevated temperature promoted a detrimental effect on MPB biomass (using both NDVI and Chl *a* concentrations as proxies) and photosynthetic performance (through the quantification of maximum photosynthetic electron transport efficiency and light utilization coefficient by PAM fluorometry). This effect was recorded under both control and elevated CO_2_. Using a non-tidal mesocosm system solely for 7 days, Hicks et al. [11] also found lower MPB biomass at higher temperatures for a mudflat of the Ythan estuary in Scotland at three levels of atmospheric CO_2_ concentrations. On the other hand, Torstensson et al. [37] found that biomass and photosynthetic activity of the benthic/sea ice diatom *Navicula directa* were promoted by elevated temperature. However, the relevant temperatures tested in the latter 7-day laboratory study were 0.5 and 4.5°C.

Intertidal MPB communities are exposed to extremely high temperature fluctuations in their natural environment. In the Tagus estuary, if emersion coincides with summer midday, the exposed dark-coloured mudflat sediment surface can reach temperatures above 30°C [20], clearly exceeding the higher temperature tested in this study. On the other hand, sediment temperature drops to a mean temperature of 18°C during summer immersion periods [20]. Hence, MPB seem to be able to cope with extremely high temperature fluctuations and short periods of very high temperature exposure. On the other hand, this study indicates that there is a significant effect on the MPB community when a less pronounced but prolonged increase in sediment temperature is applied. It is legitimate to assume that the productive potential of MPB present in the temperate Tagus estuary intertidal system may be negatively impacted by higher temperatures in the future.

Elevated temperature had also significant effects on the composition of the MPB community, causing a change on the relative abundance of major groups of microalgae. While diatoms were dominant in all treatments, higher temperature led to an increase in the relative abundance of cyanobacteria. It has been previously observed that cyanobacteria can be favoured over diatoms at higher temperatures in mixed benthic biofilms [38]. Furthermore, higher temperature also affected the relative abundance of major benthic diatom species present in the MPB community. Temperature-driven changes in the dynamics of phototrophic and heterotrophic organisms of a typically mixed benthic community are also expected to occur. Previous studies on diatom-dominated MPB of intertidal and subtidal systems showed that an increase in temperature stimulates more heterotrophy than photosynthetic activity, thus leading to a heterotrophic-dominated benthic community under elevated temperatures [9,10]. Hence, a noticeable change in the structure of the MPB community of the Tagus estuary intertidal system can be expected to occur under higher temperatures promoted by climate change.

Elevated CO_2_ and higher temperature led to a transient (day 6) increase in *ETR*_max_, as rates of light-saturated photosynthesis are generally limited by carbon metabolism (namely fixation by ribulose-1,5-bisphosphate carboxylase/oxygenase, RUBISCO) [39]. However, by the end of the experimental period, elevated CO_2_ had a beneficial effect on MPB biomass only at the lower temperature tested and when considering Chl *a* as biomass proxy. No significant effects of CO_2_ were detected on the relative abundance of major groups of microalgae and benthic diatom species.

To maintain efficient photosynthetic rates under limited CO_2_ supply, diatoms have developed high efficiency carbon concentrating mechanisms (CCMs) e.g. [12,13]. As these mechanisms grant full saturation of RUBISCO catalytic centres it is generally assumed that diatom photosynthesis is not limited by dissolved inorganic carbon availability. Accordingly, Hicks et al. [11] found no significant increase on MPB biomass of muddy intertidal sediments under increased CO_2_ levels. Surprisingly, Torstensson et al. [37] reported that *N. directa* was negatively affected by CO_2_ enrichment, although the mechanism causing this effect was not identified. On the other hand, examining the colonisation of artificial substrata across a natural CO_2_ gradient, Johnson et al. [40] found that elevated CO_2_ increased microphytobenthos biomass and induced diatom community shifts by promoting the growth of large pennate species. The latter authors argued that some diatoms could optimise resource allocation, benefiting from increasing CO_2_ through a reduction in the energy costs of their CCMs.

Further challenging the notion of CO_2_-insentitive photosynthesis in diatoms, Admiraal et al. [14] provided indirect experimental evidence of inorganic carbon limitation in benthic diatom mats cultured in the laboratory. In ^14^C tracer column experiments, Cook and Roy [15] also found that increased rates of pore-water advection or addition of HCO_3_^−^ increased photosynthesis to similar rates in MPB of subtidal sandy sediments. Again, the supply of HCO_3_^−^ was found to increase photosynthetic rates of highly productive MPB natural communities of intertidal muddy sediments (Vieira S., unpublished data).

The beneficial effect of elevated CO_2_ on MPB biomass at the lower temperature tested in our study suggests that carbon may have become a limiting resource for the MPB community. Upward migration of diatom cells to the sediment surface occurs in this benthic community during diurnal low tides, leading to the formation of an extremely dense biofilm in a relatively thin photic layer (the first hundreds of micrometers) [41]. In this crowded community, carbon may be a limiting resource even for organisms with high efficiency CCMs.

## Conclusions

As MPB are the main primary producers of many intertidal and shallow subtidal environments, changes in MPB biomass will certainly impact the trophodynamics of these systems. Nonetheless, very few studies have considered the interactive effects of climate change variables on MPB communities [11]. There are obvious limitations in providing realistic interpretations of natural ecosystem response by using artificial systems such as the one used in this study. For example, longer-term increased temperature could favor selection and growth of high-temperature adapted MPB species, that could partialy modulate the observed negative impact on biomass and productivity. Nevertheless, small-scale experiments in microcosms or mesocosms can provide valuable insights on how complex ecosystems will cope with climate change [42]. In this work, elevated temperatures under both present day and increased CO_2_ led to a reduction of MPB biomass and photosynthetic performance, an increase of cyanophytes and a change in the relative abundance of major benthic diatom species present. Overall, it suggests that the interactive effects of studied parameters could have a detrimental impact on the structure and productivity of intertidal MPB, and eventually in related ecosystem services.

## Additional files

Additional file 1: Figure S1.
**Experimental life support system (ELSS). Photographs of the flow-through experimental life support system (ELSS) used in this study.** General view of the ELSS (**A**); Approximation showing two microcosms with the sediment surface cover by MPB and the pipe system for tidal water in and outflow (**B**). For more details see Coelho et al. [23]. Additional file 2: Table S1. Species composition and relative abundance of benthic diatoms under control and elevated CO_2_ and temperature. Diatom composition and relative abundance (%, mean ± standard error, n = 4) of a Tagus estuary intertidal microphytobenthos community at the beginning of the experiment (T0) and after 11 days under different temperatures and pH. T 18°C, pH 8.0: Temperature = 18°C, pH = 8.0; T 24°C, pH 8.0.Temperature = 24°C, pH = 8.0; T 18°C, pH 7.4.Temperature = 18°C, pH = 7.4; T 24°C, pH 7.4: Temperature = 24°C, pH = 7.4.

## Abbreviations

CCMs: Carbon concentrating mechanisms
Chl *a*: Chlorophyll *a*
ELSS: Experimental life support system
*E*_k_: Light saturation parameter
*α*: Light utilization coefficient
*F*_v_/*F*_m_: Maximum quantum yield of photosystem II
*ETR*_max_: Maximum relative electron transport rate
MPB: Microphytobenthos
NDVI: Normalized difference vegetation index
PAM: Pulse amplitude modulated
RLC: Rapid light-response curves
