# Supplementary figures and images for: Effects of elevated temperature and CO2 on intertidal microphytobenthos
Source: BMC Ecol. 2015 Apr 1;15:10. doi: 10.1186/s12898-015-0043-y (PMC4411721; doi:10.1186/s12898-015-0043-y)

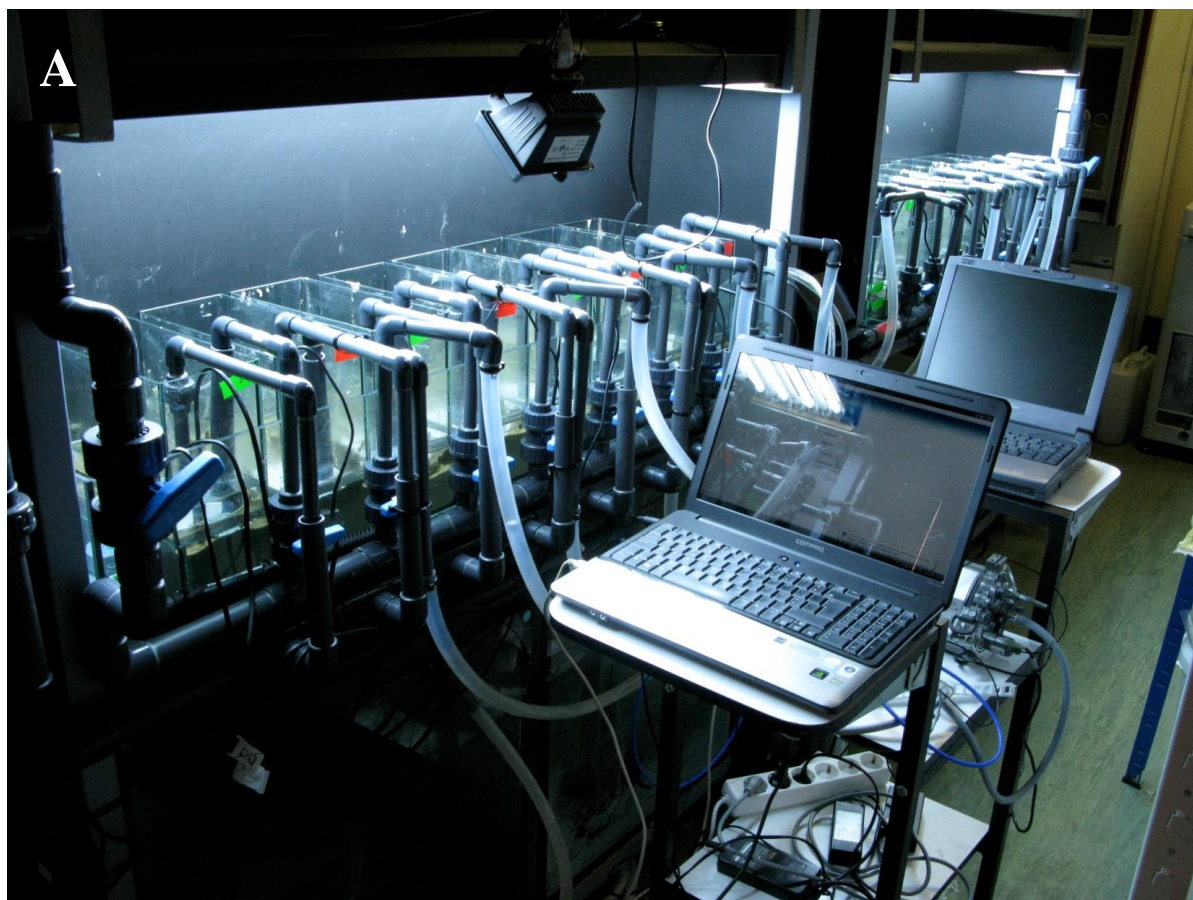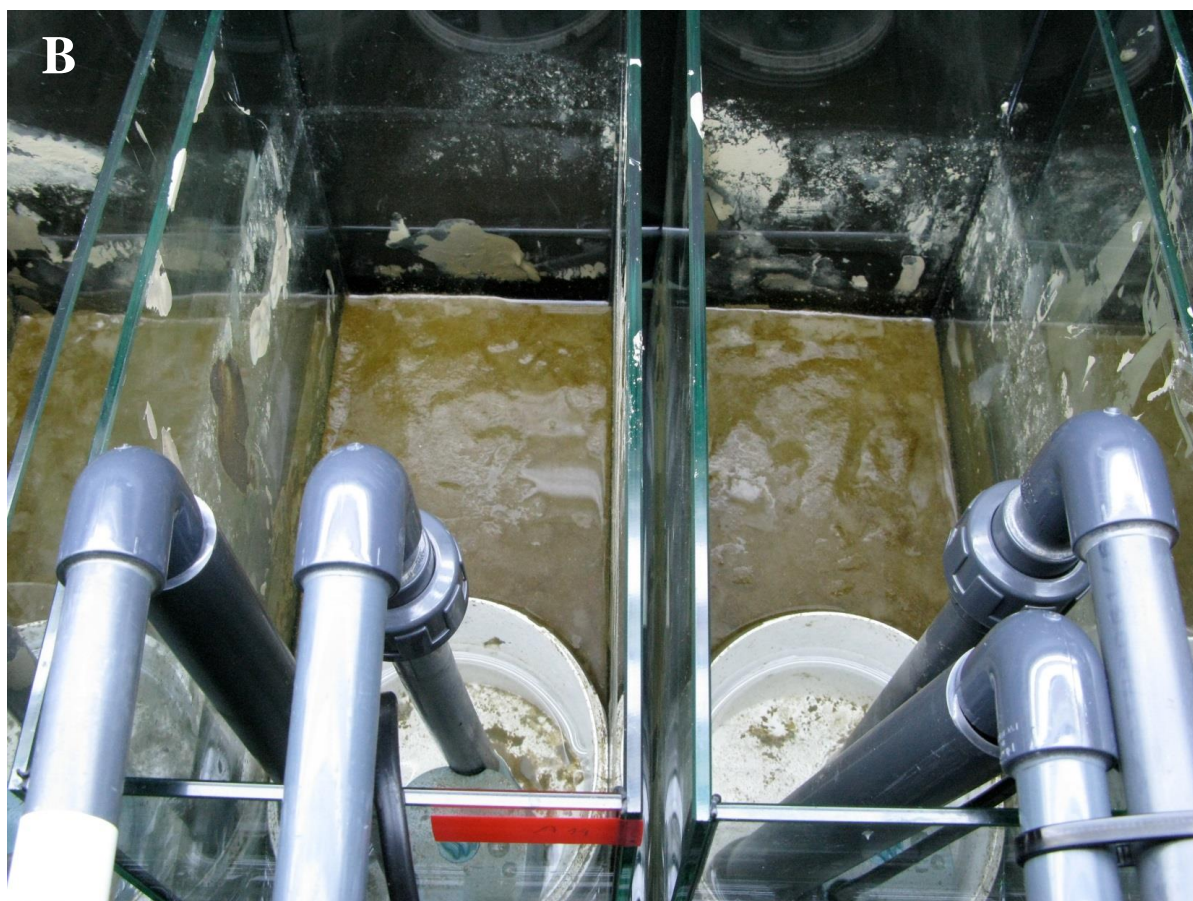

Supplement: Additional file 1: Figure S1. — Experimental life support system (ELSS). Photographs of the flow-through experimental life support system (ELSS) used in this study. General view of the ELSS (A); Approximation showing two microcosms with the sediment surface cover by MPB and the pipe system for tidal water in and outflow (B). For more details see Coelho et al. [23]. [file 12898_2015_43_MOESM1_ESM.pdf]
