# Supplementary material for: Effects of elevated temperature and CO2 on intertidal microphytobenthos
Source: BMC Ecol. 2015 Apr 1;15:10. doi: 10.1186/s12898-015-0043-y (PMC4411721; doi:10.1186/s12898-015-0043-y)
Supplement: Additional file 2: Table S1. — Species composition and relative abundance of benthic diatoms under control and elevated CO2 and temperature. Diatom composition and relative abundance (%, mean ± standard error, n = 4) of a Tagus estuary intertidal microphytobenthos community at the beginning of the experiment (T0) and after 11 days under different temperatures and pH. T 18°C, pH 8.0: Temperature = 18°C, pH = 8.0; T 24°C, pH 8.0.Temperature = 24°C, pH = 8.0; T 18°C, pH 7.4.Temperature = 18°C, pH = 7.4; T 24°C, pH 7.4: Temperature = 24°C, pH = 7.4. [file 12898_2015_43_MOESM2_ESM.docx]

**Table S1.** **Species composition and relative abundance of microphytobenthic diatoms under control and elevated CO_2_ and temperature.** Diatom composition and relative abundance (%, mean ± standard error, n=4) of a Tagus estuary intertidal microphytobenthic community at the beginning of the experiment (T0) and after 11 days under different temperatures and pH. T 18°C pH 8.0: Temperature = 18°C, pH = 8.0; T 24°C pH 8.0.Temperature = 24°C, pH = 8.0; T 18°C pH 7.4.Temperature = 18°C, pH = 7.4; T 24°C pH 7.4: Temperature = 24°C, pH=7.4.

| **Species** | **T0** | **T 18ºC**  **pH 8.0** | **T 18ºC**  **pH 7.4** | **T 24ºC**  **pH 8.0** | **T 24ºC**  **pH 7.4** |
| --- | --- | --- | --- | --- | --- |
| *Achnanthes lemmermannii* var. *obtusa* Hustedt |  |  | 0.04 (0.04) |  |  |
| *Achnanthes* s.l. sp.1 |  |  |  |  | 0.11 (0.11) |
| *Achnanthes* s.l. sp.2 |  | 0.04 (0.04) |  |  | 0.20 (0.13) |
| *Achnanthidium minutissimum* (Kützing) Czarnecki |  |  | 0.16 (0.16) | 0.10 (0.10) | 0.05 (0.05) |
| *Actinocyclus normanii* (Gregory) Hustedt | 0.08 (0.08) | 0.04 (0.04) | 0.10 (0.10) | 0.16 (0.10) | 0.09 (0.09) |
| *Amphora arenicola* Grunow in Cleve |  |  | 0.04 (0.04) |  |  |
| *Amphora* cf. *helenensis* Giffen |  |  |  |  | 0.05 (0.05) |
| *Amphora* cf. *micrometra* Giffen |  | 0.13 (0.13) | 0.18 (0.18) |  |  |
| *Amphora* cf. *pediculus* (Kützing) Grunow in Schmidt et al. | 0.08 (0.08) | 0.13 (0.08) | 0.10 (0.10) | 0.20 (0.11) | 0.37 (0.32) |
| *Amphora* cf. *subacutiuscula* Schoeman | 0.31 (0.20) | 0.27 (0.14) | 0.32 (0.13) | 0.72 (0.41) | 0.33 (0.22) |
| *Amphora* cf. *tenuissima* Hustedt |  |  |  | 0.43 (0.16) | 0.29 (0.16) |
| *Aulacoseira granulata* (Ehrenberg) Simonsen | 0.24 (0.24) |  |  | 0.11 (0.11) |  |
| *Aulacoseira islandica* (O. Müller) Simonsen |  |  | 0.12 (0.12) |  |  |
| *Aulacoseira subartica* (O. Müller) Haworth |  |  |  | 0.10 (0.06) |  |
| *Berkeleya rutilans* (Trentepohl ex Roth) Grunow |  |  |  | 0.05 (0.05) |  |
| *Biremis lucens* (Hustedt) Sabbe Witkowski & Vyverman |  | 0.09 (0.06) |  | 0.30 (0.17) | 0.15 (0.09) |
| *Catenula adhaerens* (Mereschkowsky) Mereschkowsky |  | 0.04 (0.04) |  | 0.24 (0.24) | 0.14 (0.14) |
| *Catenula* sp.1 |  |  |  | 0.05 (0.05) | 0.09 (0.09) |
| *Catenula* sp.2 |  |  |  |  | 0.09 (0.09) |
| *Climaconeis fasciculata* (Grunow ex Cleve) Cox |  | 0.03 (0.03) |  |  | 0.09 (0.05) |
| *Cocconeiopsis breviata* (Hustedt) Witkowski. Lange-Bertalot & Metzeltin | 0.08 (0.08) | 0.06 (0.06) |  | 0.21 (0.09) | 0.14 (0.14) |
| *Cocconeis* cf. *placentula* Ehrenberg |  |  | 0.04 (0.04) | 0.05 (0.05) | 0.05 (0.05) |
| *Cocconeis hauniensis* Witkowski emend. Witkowski |  | 0.13 (0.05) |  | 0.14 (0.09) | 0.19 (0.19) |
| *Cocconeis pelta* Schmidt |  |  | 0.04 (0.04) | 0.10 (0.10) | 0.09 (0.05) |
| *Cocconeis peltoides* Hustedt |  |  |  | 0.19 (0.19) | 0.14 (0.14) |
| *Cocconeis scutellum* Ehrenberg var. *parva* (Grunow) Cleve in Van Heurck |  |  |  |  | 0.05 (0.05) |
| *Coscinodiscus radiatus* Ehrenberg |  |  | 0.05 (0.05) |  |  |
| *Cyclotella atomus* Hustedt |  |  |  |  | 0.09 (0.09) |
| *Cyclotella meneghiniana* Kützing | 0.08 (0.08) | 0.10 (0.06) | 0.09 (0.05) |  | 0.14 (0.09) |
| *Cymatosira belgica* Grunow in Van Heurck |  | 0.18 (0.10) |  |  | 0.56 (0.56) |
| *Neodelphineis pelagica* Takano |  | 0.04 (0.04) |  |  |  |
| *Dickieia* sp.1 | 0.15 (0.27) |  |  |  |  |
| *Diploneis didyma* (Ehrenberg) Cleve | 0.08 (0.14) |  | 0.06 (0.06) |  |  |
| *Eolimna minima* (Grunow in Van Heurck) Lange-Bertalot |  |  |  | 0.05 (0.05) |  |
| *Fallacia* cf. *teneroides* (Hustedt) Mann |  |  |  |  | 0.09 (0.09) |
| *Fallacia florinae* (Moeller) Witkowski | 0.08 (0.13) | 0.03 (0.03) | 0.04 (0.04) | 0.19 (0.13) | 0.19 (0.19) |
| *Fallacia oculiformis* (Hustedt) Mann |  |  |  | 0.19 (0.13) |  |
| *Frustulia interposita* (Lewis) De Toni | 0.78 (0.58) | 1.39 (0.25) | 1.51 (0.35) | 0.93 (0.29) | 1.63 (0.61) |
| *Gyrosigma acuminatum* (Kützing) Rabenhorst | 8.88 (3.72) | 5.69 (0.84) | 5.63 (0.35) | 2.09 (0.47) | 3.29 (0.52) |
| *Gyrosigma fasciola* (Ehrenberg) Griffith & Henfrey | 0.31 (0.35) | 0.04 (0.04) | 0.44 (0.24) | 0.20 (0.11) | 0.18 (0.11) |
| *Gyrosigma* cf. *limosum* Sterrenburg & Underwood | 6.04 (1.12) | 1.36 (0.54) | 1.42 (0.20) | 1.27 (0.30) | 2.89 (0.85) |
| *Gyrosigma scalproides* (Rabenhorst) Cleve |  | 0.04 (0.04) |  |  |  |
| *Gyrosigma* sp.1 | 1.17 (0.81) | 1.60 (0.26) | 0.73 (0.19) | 0.97 (0.35) | 0.61 (0.15) |
| *Halamphora* cf. *abuensis* (Foged) Levkov |  |  |  | 0.19 (0.13) |  |
| *Halamphora* sp.1 |  |  | 0.05 (0.05) | 0.05 (0.05) | 0.05 (0.05) |
| *Hippodonta caotica* Witkowski. Lange-Bertalot & Metzeltin | 0.08 (0.13) | 0.07 (0.04) |  | 0.05 (0.05) | 0.04 (0.04) |
| *Luticola mutica* (Kützing) Mann. |  | 0.04 (0.04) |  |  |  |
| *Minidiscus chilensis* Rivera in Rivera & Koch | 6.53 (3.38) | 6.22 (1.25) | 5.31 (1.66) | 12.64 (2.89) | 15.69 (1.34) |
| *Navicula abscondita* Hustedt |  |  |  |  | 0.05 (0.05) |
| *Navicula* cf. *aleksandrae* Lange-Bertalot. Bogaczewicz-Adamczak & Witkowski | 0.08 (0.13) |  |  |  | 0.41 (0.15) |
| *Navicula arenaria* Donkin |  |  |  | 0.09 (0.09) |  |
| *Navicula bozenae* Lange-Bertalot. Witkowski & Zgrundo |  | 0.21 (0.21) | 0.16 (0.10) | 0.23 (0.17) | 0.14 (0.09) |
| *Navicula diserta* Hustedt |  | 0.12 (0.08) | 0.17 (0.11) | 0.05 (0.05) | 0.19 (0.13) |
| *Navicula flagellifera* Hustedt |  |  |  | 0.05 (0.05) |  |
| *Navicula gregaria* Donkin m.1 | 10.98 (0.54) | 5.26 (0.51) | 5.96 (0.63) | 7.21 (1.77) | 6.25 (1.30) |
| *Navicula* cf. *microdigitoradiata* Lange-Bertalot |  |  | 0.14 (0.14) | 0.09 (0.05) |  |
| *Navicula* cf. *mollis* (W. Smith) Cleve |  |  |  |  | 0.10 (0.06) |
| *Navicula paeninsulae* Cholnoky |  | 0.04 (0.04) |  |  | 0.05 (0.05) |
| *Navicula pargemina* Underwood & Yallop | 0.15 (0.27) | 0.46 (0.16) | 0.20 (0.15) | 2.14 (0.85) | 0.67 (0.53) |
| *Navicula* cf. *phyllepta* Kützing | 8.22 (3.44) | 6.20 (0.87) | 5.65 (0.87) | 12.28 (1.74) | 10.67 (2.02) |
| *Navicula platyventris* Meister |  |  |  | 0.05 (0.05) |  |
| *Navicula ponticula* Giffen |  | 0.04 (0.04) |  |  |  |
| *Navicula salinarum* Grunow |  |  |  |  | 0.23 (0.23) |
| *Navicula* cf. *salinicola* Hustedt | 0.16 (0.28) |  |  |  |  |
| *Navicula recens* (Lange-Bertalot) Lange-Bertalot | 0.15 (0.27) | 0.06 (0.06) | 0.04 (0.04) | 0.10 (0.06) | 0.05 (0.05) |
| *Navicula spartinetensis* Sullivan & Reimer | 37.33 (7.18) | 51.66 (2.04) | 55.02 (2.04) | 23.09 (5.89) | 20.85 (3.85) |
| *Navicula viminoides* Giffen |  |  |  |  | 0.23 (0.23) |
| *Navicula* sp.3 | 0.31 (0.35) | 0.50 (0.15) | 0.44 (0.20) |  |  |
| *Nitzschia* cf. *aequorea* Hustedt | 3.90 (1.45) | 6.55 (0.81) | 6.85 (2.17) | 9.55 (3.79) | 10.90 (2.85) |
| *Nitzschia* cf. *aurariae* Cholnoky | 2.35 (1.72) | 0.76 (0.31) | 0.57 (0.24) | 7.58 (3.65) | 4.21 (0.85) |
| *Nitzschia* cf. *dissipata* (Kützing) Grunow | 0.93 (0.26) |  | 0.22 (0.17) | 0.55 (0.21) | 0.44 (0.10) |
| *Nitzschia* cf. *distans* Gregory | 0.08 (0.13) | 0.68 (0.26) | 0.56 (0.23) | 0.05 (0.05) | 0.13 (0.13) |
| *Nitzschia* cf. *parvula* W. Smith non Lewis | 0.70 (0.41) | 0.14 (0.09) | 0.47 (0.27) | 0.57 (0.23) | 0.66 (0.39) |
| *Nitzschia frustulum* (Kützing) Grunow in Cleve & Grunow | 0.23 (0.23) | 0.38 (0.19) | 0.17 (0.11) | 0.58 (0.25) | 0.48 (0.18) |
| *Nitzschia navicularis* (Brébisson) Grunow |  | 0.06 (0.06) |  |  |  |
| *Nitzschia sigma* (Kützing) W. Smith | 0.23 (0.01) | 0.20 (0.08) | 0.52 (0.14) | 0.34 (0.16) | 1.26 (0.50) |
| *Nitzschia* cf. *tubicola* Grunow in Cleve *&* Grunow |  |  | 0.05 (0.05) |  |  |
| *Nitzschia valdestriata* Aleem & Hustedt |  | 0.15 (0.11) | 0.05 (0.05) | 0.29 (0.12) | 0.11 (0.11) |
| *Nitzschia* sp.1 |  |  |  | 0.05 (0.05) |  |
| *Nitzschia* sp.4 | 0.31 (0.35) |  |  | 0.05 (0.05) | 0.16 (0.10) |
| *Nitzschia* sp.5 |  | 0.23 (0.11) | 0.04 (0.04) |  |  |
| *Nitzschia* sp.6 |  | 0.16 (0.12) | 0.32 (0.13) | 0.59 (0.29) | 0.28 (0.16) |
| *Nitzschia* sp.7 |  |  |  | 0.05 (0.05) |  |
| *Nitzschia* sp.8 |  |  | 0.10 (0.06) | 0.19 (0.11) |  |
| *Nitzschia* sp.9 |  |  |  |  | 0.05 (0.05) |
| *Nitzschia* sp.10 |  |  |  |  | 0.16 (0.16) |
| *Nitzschia* sp.11 | 0.47 (0.48) | 0.13 (0.13) |  | 1.07 (0.36) |  |
| *Nitzschia* sp.12 |  | 0.21 (0.21) | 0.06 (0.06) |  | 0.26 (0.26) |
| *Opephora guenter-grassii* (Witkowski & Lange-Bertalot) Sabbe & Vyverman |  |  |  | 0.10 (0.10) |  |
| *Opephora* sp.1 |  |  |  | 0.05 (0.05) |  |
| *Parlibellus berkeleyi* (Kützing) Cox |  |  | 0.10 (0.06) |  |  |
| *Petrodictyon gemma* (Ehrenberg) Mann in Round. Crawford & Mann | 0.30 (0.26) | 0.36 (0.15) | 0.10 (0.10) | 0.35 (0.10) | 0.05 (0.05) |
| *Pierrecomperia catenuloides* Sabbe. Vyverman & Ribeiro |  | 0.08 (0.08) | 0.05 (0.05) | 0.09 (0.09) |  |
| *Plagiogrammopsis minima* (Salah) Sabbe & Witkowski | 0.16 (0.28) | 0.89 (0.36) | 0.57 (0.32) | 1.37 (0.33) | 2.02 (0.23) |
| *Plagiotropis vanheurckii* Grunow in Van Heurck | 3.96 (0.58) | 2.12 (0.27) | 1.13 (0.18) | 0.64 (0.23) | 1.23 (0.59) |
| *Planothidium* cf. *lemmermannii* Hustedt (Morales) | 0.08 (0.13) |  |  |  | 0.05 (0.05) |
| *Planothidium delicatulum* s.l. (Kützing) Round & Bukhtiyarova m.1 |  | 0.08 (0.08) | 0.05 (0.05) | 0.24 (0.09) | 0.29 (0.05) |
| *Planothidium delicatulum* s.l. (Kützing) Round & Bukhtiyarova m.2 | 0.08 (0.13) | 0.13 (0.05) | 0.10 (0.06) | 0.05 (0.05) | 0.14 (0.09) |
| *Planothidium delicatulum* s.l. (Kützing) Round & Bukhtiyarova m.3 | 0.08 (0.14) | 0.04 (0.04) |  | 0.05 (0.05) |  |
| *Planothidium deperditum* (Giffen) Witkowski. Lange-Bertalot & Metzeltin |  |  |  | 0.10 (0.10) | 0.05 (0.05) |
| *Pleurosigma* sp.1 | 0.31 (0.14) | 0.32 (0.08) | 0.29 (0.05) |  | 0.28 (0.12) |
| *Psammodictyon panduriforme* var. *continuum* (Grunow) Snoeijs |  | 0.03 (0.03) |  | 0.16 (0.10) |  |
| *Reimeria sinuata* (Gregory) Kociolek & Stoermer |  |  |  |  | 0.05 (0.05) |
| *Seminavis* sp. 1 |  |  |  |  | 0.05 (0.05) |
| *Staurophora salina* (W. Smith) Mereschkowsky | 0.38 (0.47) | 0.50 (0.20) |  | 0.26 (0.13) | 0.46 (0.20) |
| *Stephanodiscus rotula* (Kützing) Hendey | 0.08 (0.13) |  |  |  |  |
| *Surirella atomus* Hustedt | 0.24 (0.24) | 0.24 (0.10) | 0.17 (0.10) | 0.74 (0.21) | 0.34 (0.10) |
| *Surirella curvifacies* Brun |  | 0.20 (0.11) | 0.08 (0.08) | 0.14 (0.14) |  |
| *Surirella* sp.1 | 0.16 (0.28) | 0.06 (0.06) |  | 0.05 (0.05) | 0.05 (0.05) |
| *Thalassiocyclus lucens* (Hustedt) Håkansson & Mahood | 0.16 (0.28) | 0.16 (0.09) | 0.05 (0.05) | 0.33 (0.20) | 0.59 (0.25) |
| *Thalassiosira angulata* (Gregory) Hasle | 0.15 (0.26) |  | 0.08 (0.05) | 0.10 (0.06) | 0.04 (0.04) |
| *Thalassiosira binata* Fryxell | 0.08 (0.14) |  |  |  |  |
| *Thalassiosira* cf. *profunda* (Hendey) Hasle | 0.08 (0.13) |  |  |  | 0.04 (0.04) |
| *Thalassiosira minima* Gaarder | 0.24 (0.42) | 0.14 (0.09) | 0.22 (0.08) | 0.56 (0.33) | 0.35 (0.26) |
| *Thalassiosira proschkinae* Makarova in Makarova. Genkal & Kuzmln | 0.62 (0.70) | 0.72 (0.47) | 0.86 (0.67) | 0.83 (0.37) | 0.72 (0.27) |
| *Thalassiosira* cf. *pseudonana* Hasle & Heimdal | 1.24 (0.17) | 1.75 (0.20) | 1.92 (0.65) | 4.93 (1.44) | 5.89 (2.47) |
| *Thalassiosira* sp.1 | 0.08 (0.13) |  |  |  |  |
| *Thalassiosira* sp.2 |  |  |  | 0.05 (0.05) |  |
| *Tryblionella apiculata* Gregory |  | 0.17 (0.06) |  |  | 0.14 (0.09) |
